# BETAV: A Unified BEV-Transformer and Bézier Optimization Framework for Jointly Optimized End-to-End Autonomous Driving

**DOI:** 10.3390/s25113336

**Published:** 2025-05-26

**Authors:** Rui Zhao, Ziguo Chen, Yuze Fan, Fei Gao, Yuzhuo Men

**Affiliations:** 1 College of Automotive Engineering, Jilin University, Changchun 130025, China; rzhao@jlu.edu.cn (R.Z.); chenzg24@mails.jlu.edu.cn (Z.C.); fanyz23@mails.jlu.edu.cn (Y.F.); 2 National Key Laboratory of Automotive Chassis Integration and Bionics, Jilin University, Changchun 130025, China; 3 School of Mechanical and Electrical Engineering, Suqian University, Suqian 223800, China; menyuzhuo@squ.edu.cn

**Keywords:** autonomous driving, transformer, bird’s eye view, Bézier curves, trajectory planning

## Abstract

End-to-end autonomous driving demands precise perception, robust motion planning, and efficient trajectory generation to navigate complex and dynamic environments. This paper proposes BETAV, a novel framework that addresses the persistent challenges of low 3D perception accuracy and suboptimal trajectory smoothness in autonomous driving systems through unified BEV-Transformer encoding and Bézier-optimized planning. By leveraging Vision Transformers (ViTs), our approach encodes multi-view camera data into a Bird’s Eye View (BEV) representation using a transformer architecture, capturing both spatial and temporal features to enhance scene understanding comprehensively. For motion planning, a Bézier curve-based planning decoder is proposed, offering a compact, continuous, and parameterized trajectory representation that inherently ensures motion smoothness, kinematic feasibility, and computational efficiency. Additionally, this paper introduces a set of constraints tailored to address vehicle kinematics, obstacle avoidance, and directional alignment, further enhancing trajectory accuracy and safety. Experimental evaluations on Nuscences benchmark datasets and simulations demonstrate that our framework achieves state-of-the-art performance in trajectory prediction and planning tasks, exhibiting superior robustness and generalization across diverse and challenging Bench2Drive driving scenarios.

## 1. Introduction

Autonomous driving technology has recently become a critical area of research in intelligent transportation and future mobility, gradually transitioning from conceptualization to practical application. Over the past few decades, autonomous driving technology has achieved remarkable advancements, leading to the development of a well-established layered architecture comprising multiple modules, including perception, path planning, decision-making, and control [1]. Despite their success, traditional hierarchical control systems face inherent limitations [2]. These systems rely heavily on meticulously designed intermediate interfaces between modules and require detailed modeling of the environment. Consequently, their performance is highly dependent on the accuracy and seamless integration of each submodule. Any inaccuracies or inconsistencies in one module can propagate through the pipeline, potentially compromising the overall system’s reliability and robustness in real-world scenarios [3,4,5,6,7].

End-to-end autonomous driving [8,9,10,11] has recently emerged as a promising direction in the field, directly mapping raw sensor data (e.g., cameras and LiDAR) to decision outputs via deep learning models. This learning-based paradigm minimizes reliance on handcrafted rules and error accumulation across traditional modules while unifying perception, planning, and control. The recent advancements in deep learning and optimization have enhanced its efficiency, decision-making speed, and robustness in real-world autonomous driving. The recent research [3,11,12] in the field of end-to-end autonomous driving primarily focuses on two core modules: (1) feature encoding in the perception module to ensure that the model can accurately and efficiently understand complex driving environments, and (2) trajectory generation and control policy synthesis directly from latent representations, enabling the model to translate scene understanding into executable driving actions.

In terms of perception encoding, common approaches like Convolutional Neural Networks (CNNs) and Vision Transformers (ViTs) extract features from sensor data (e.g., camera images and LiDAR) for direct input into decision-making modules. This approach [8,13,14] bypasses intermediate tasks such as object tracking and traffic semantic understanding, directly mapping sensor inputs to control outputs, mimicking the “perception-to-decision” mechanism of human driving. While this simplifies the model architecture, it faces challenges such as high training complexity, dependence on large-scale datasets, and limited interpretability, reducing robustness in complex or unseen scenarios [15]. Another widely adopted approach [8,10,16] incorporates intermediate tasks in scene perception. This method first encodes sensor data into features and decodes them into high-level semantic information (e.g., positions of traffic agents and lane boundaries) for planning and control. Typical tasks include object detection, semantic segmentation, or multi-task combinations, offering advantages like greater interpretability and reduced learning complexity for control modules [17]. Leveraging models such as YOLO [18] or CenterNet [19], this type of method improves system performance. However, they face inherent limitations in reliably estimating precise real-world coordinates and the dynamic states of traffic agents [20]. These challenges stem from the difficulty in inferring accurate depth and motion information from 2D visual data, particularly in scenarios involving occlusions, overlapping objects, or complex interactions in the 3D environment.

In terms of planning modules, the mainstream approaches can be categorized into two types [21,22]: (1) directly outputting low-level control signals for the vehicle, such as throttle, brake, and steering angle, and (2) generating trajectory planning results. From a conceptual perspective, the first approach aligns more closely with the philosophy of end-to-end autonomous driving as it directly maps perception to control [15]. However, this method imposes strict real-time requirements on the model and is often limited by hardware differences across vehicles (e.g., variations in powertrain response and steering ratios), which reduces its adaptability and broad applicability [11]. The second method employs a hierarchical control architecture, where a neural network model serves as the upper layer to generate multi-time-step trajectory planning for future sequences, while the lower layer—typically composed of conventional control algorithms (e.g., PID and MPC) or enhanced approaches such as RMPC and VUF-PID—translates these trajectory plans into specific low-level control signals [12,23,24]. This separation of high-level decision-making from low-level control enhances the model’s generalizability and adaptability. However, a notable limitation of this approach is the sparsity of discrete trajectory points in the planning stage, which can lead to suboptimal trajectory smoothness and introduce challenges in accurately representing the desired path [25]. Additionally, the method heavily relies on the richness and diversity of the training dataset as a lack of variety in the data can result in poor generalization to unseen or complex driving scenarios [26].

To address the critical challenges in autonomous driving systems, including the perception module’s limitations in 3D inference accuracy, training complexity, and limited interpretability, as well as the motion planning module’s issues with real-time adaptability, trajectory smoothness, and data dependency, we propose an innovative framework that unifies advanced perception and planning techniques. For perception, we utilize Vision Transformers (ViTs) to encode visual information into a Bird’s Eye View (BEV) representation, offering interpretable and robust spatial understanding of traffic agents, including precise positions, orientations, and future trajectories. This improves 3D inference accuracy and enhances interpretability, boosting performance in complex scenarios. For planning, we introduce a novel Bézier curve-based trajectory representation, which ensures kinematic feasibility, smooth transitions, and reduced computational demand. This approach mitigates the limitations of the traditional discrete methods, enhances generalization to diverse scenarios, and improves real-time adaptability.

Our contributions can be summarized as follows:

1. Query-Based Interaction Mechanism: We introduce a novel query-based interaction mechanism that enables efficient fusion of multi-modal sensor signals, facilitating the extraction of explicit environmental features and implicit contextual cues for ViTs. This approach enhances perception accuracy while improving the safety and adaptability of autonomous navigation in complex real-world scenarios.
2. Bézier-Parameterized Trajectory Framework: We propose an innovative trajectory representation framework utilizing Bézier curves to parameterize high-dimensional outputs, transforming discrete trajectory points into a low-dimensional and continuous representation. This approach significantly reduces the network’s learning complexity while ensuring trajectory smoothness, kinematic feasibility, and adaptability across diverse driving scenarios.
3. Performance-Validated Evaluation Framework: We conducted tests on the nuScenes dataset to evaluate trajectory planning accuracy and safety while performing generalizability tests in the Carla simulator for system performance verification. The experimental results demonstrate that our method achieves superior reliability and stability in dynamic and complex scenarios compared to the state-of-the-art approaches.

## 2. Related Works

Perception and Prediction: Accurate perception of driving scenes forms the foundation and safety guarantee of autonomous driving. In end-to-end driving systems, while 2D detection provides fundamental visual cues through cross-task feature sharing, 3D detection establishes hierarchical latent representations for precise environment modeling and obstacle identification, enabling synergistic integration within a unified architecture [27]. Traditional 3D perception methods typically address 3D object detection or map segmentation tasks independently. For 3D object detection, the early methods followed the principles of 2D detection, where 3D bounding boxes were predicted based on 2D bounding boxes. DETR3D [28] introduced a novel approach by utilizing 3D queries to sample corresponding 2D image features, enabling detection without the need for non-maximum suppression (NMS). PETR [29] further advanced this by embedding 3D positional encoding into 2D image features and leveraging detection queries to extract object features through an attention mechanism [30].

More recently, BEV representations have gained significant attention and made substantial contributions to perception [31,32,33,34,35,36,37]. LSS [38] pioneered the use of depth prediction to project perspective features into the BEV space. OFT [39] and ImVoxelNet [40] utilized predefined voxels that are projected onto image features to construct voxel-based scene representations. M2BEV [41] extended BEV representations to simultaneously perform multiple perception tasks, highlighting their versatility. FIERY [32] and BEVerse [37] leveraged BEV feature maps to predict dense map segmentation, while LaneGAP [34] introduced a novel path-based representation for lane modeling, effectively preserving lane continuity and encoding traffic context for planning tasks. In this work, we use a set of BEV queries, agent queries, and map queries to complete scene perception, and further use these query features and perception results for the motion prediction and planning stages.

Planning: Some approaches [42,43] bypass intermediate stages such as perception and motion prediction, directly predicting planning trajectories or control signals. While this end-to-end paradigm is conceptually straightforward, it suffers from limited interpretability and presents challenges in optimization and reliability. To address these limitations, explicit dense cost maps have been widely adopted due to their high interpretability [8,9,44]. These cost maps are constructed from either perception and motion prediction outputs or directly learned through data-driven modules. Handcrafted rules are often employed to select the optimal trajectory with the minimum cost. However, constructing dense cost maps is computationally expensive, and the reliance on handcrafted rules introduces challenges in robustness and generalization.

Recent advancements have explored new directions for planning. UniAD [13], for instance, effectively integrates information from multiple upstream tasks to support goal-oriented planning, achieving state-of-the-art performance in perception, prediction, and planning. PlanT [45] takes the perception ground truth as the input and encodes the scene into object-level representations for trajectory planning. Despite these improvements, challenges such as trajectory smoothness and high computational complexity persist, particularly when relying on intermediate stages like perception and motion prediction. VAD [46] improves the perception and planning performance of autonomous driving by representing driving scenes in vectorized maps and agent actions. To overcome these challenges, alternative methods have been proposed. Zhichao Han et al. [47] utilized the property of differential flatness to simplify the trajectory planning problem, significantly improving computational efficiency and trajectory smoothness. Similarly, Weihuang Chen et al. [48] introduced the feasible and smooth tree (FAST) method, which ensures both trajectory continuity and kinematic feasibility by leveraging a tree-structured planning approach. VADv2 [49] leverages human-like driving policies acquired from extensive large-scale driving demonstrations, producing a probabilistic action distribution that facilitates superior planning and driving performance.

In this work, we adopt Bézier curves to assist trajectory planning, leveraging their compact and parameterized representation. This approach substantially reduces the trajectory prediction dimensionality while inherently ensuring motion smoothness, kinematic feasibility, and computational efficiency.

## 3. Methods

**Figure 1:**
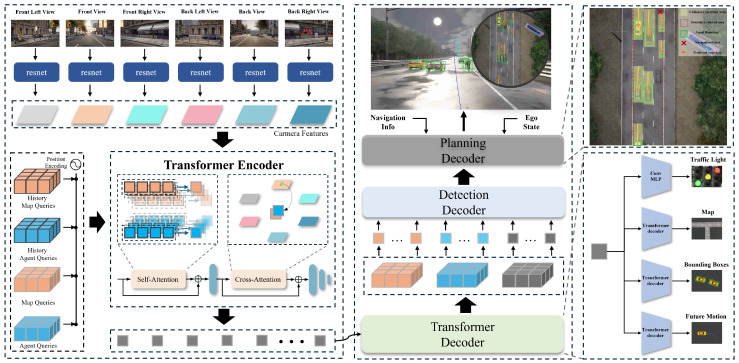
BETAV architecture. Multi-view camera features extracted by ResNet are fused with spatiotemporal embeddings via self-attention (temporal) and cross-attention (spatial) in the transformer encoder. Task-specific decoders then generate traffic agent predictions and Bézier-parameterized trajectories for safe autonomous navigation.

This work presents an end-to-end autonomous driving architecture (Figure 1) composed of two core components: (1) a perception and prediction module, which extracts high-level semantic information about the driving environment, and (2) a Bézier curve-based trajectory planning module, designed to generate smooth, kinematically feasible trajectories that enable the ego vehicle to navigate safely, legally, and compliantly with traffic regulations. The subsequent sections provide a detailed description of the problem formulation and the design of each module.

### 3.1. Problem Setting

This work aims to design an autonomous driving system that generates continuous trajectory outputs for steering and acceleration/deceleration control based on perception data obtained from onboard sensors. The sensor inputs consist of six RGB images captured from multiple perspectives and ego-vehicle states, including speed, acceleration, and GPS position. Building upon these multi-modal inputs, our methodology employs imitation learning (IL) as the foundational training paradigm, incorporating a composite loss function L formed through weighted combinations of multiple loss terms specifically designed to address distinct aspects of driving behavior. This integrated framework enables the system to generate kinematically feasible and smooth trajectories for autonomous navigation.

**Imitation Learning in Autonomous Driving:** IL, also known as learning from demonstrations, is a foundational paradigm in machine learning that allows an agent to learn desired behaviors by mimicking expert demonstrations. In the context of autonomous driving, IL provides a mechanism for vehicles to acquire complex driving behaviors by utilizing data collected from human drivers, thereby eliminating the need for explicitly programming every driving decision. The IL framework typically involves training a policy π, which maps observations *s* (e.g., raw sensor data or environment states) to actions *a* (e.g., trajectory). The objective is to minimize the discrepancy between the agent’s predicted actions and those performed by an expert using a supervised approach based on a dataset of state–action pairs (s,a). In this work, the policy π is trained in imitation learning using the collected data D with the loss function L:

(1) $$\arg\min_{\pi }E_{(s,a)∼D}\left[L(a,\pi (s))\right]$$

### 3.2. Input and Output Parameterization

**Input Representation:** The six RGB cameras are employed as input sensors, strategically arranged to ensure omnidirectional environmental coverage through their calibrated spatial configuration. The setup includes one forward-facing camera, two forward-facing cameras angled at ±55°, one rear-facing camera, and two rear-facing cameras angled at ±70°. All cameras, except for the rear-facing camera with a horizontal field of view (FOV) of 110°, have an FOV of 70° and a resolution of 1600×900. In addition to visual inputs, the trajectory planning module incorporates sparse navigation information and the ego vehicle’s dynamic state. It is important to note that the navigation information is provided as a discrete set of guidance points, typically spaced hundreds of meters apart, rather than a dense continuous trajectory. This sparse guidance structure reflects realistic navigation data used in autonomous driving systems and poses additional challenges for trajectory planning.

**Output Representation:** Bézier curves are implemented as parametric descriptors for trajectory prediction of the ego vehicle’s future motion *W* within the BEV coordinate framework. The trajectory points are sampled from a cubic Bézier curve defined by its control points $P_{0},P_{1},P_{2},P_{3}$. Mathematically, the trajectory *W* is expressed as

(2) $$W=\{\left(x,y\right)\;|\;\left(x,y\right)\in \mathrm{sample}\left(B(t)\right),\;t\in \left[0,1\right]\}$$

here, *t* is the curve parameter that determines the position along the Bézier curve, ranging from 0 to 1. B(t) represents Bézier curves:

(3) $$B\left(t\right)={\left(1-t\right)}^{3}P_{0}+3{\left(1-t\right)}^{2}tP_{1}+3\left(1-t\right)t^{2}P_{2}+t^{3}P_{3}$$

where $P_{0}$ represents the initial position of the ego vehicle, while $P_{1},P_{2},$ and $P_{3}$ are learnable control points that shape the curve. By sampling points along the curve, we generate a smooth and kinematically feasible trajectory for the ego vehicle in the BEV space.

### 3.3. Perception and Prediction Module

The image data captured by vehicle-mounted cameras are inherently sparse and low-level, posing significant challenges for direct use in planning and decision-making tasks. To address this limitation, a differentiated encoder–decoder architecture is implemented to encode raw image data into BEV-aligned high-level semantic representations.

Specifically, a set of grid-shaped learnable parameters $Q_{t}\in R^{H\times W}$ are instantiated as queries for BEVFormer, where *H* and *W* represent the spatial dimensions of the BEV plane. Each query $Q_{p}\in R$ at position $p=(x_{bq},y_{bq})$ corresponds to a specific grid cell region in the BEV plane, with each grid cell covering a real-world area of $s_{bev}$ meters. By default, the BEV feature is centered on the location of the ego vehicle, and the grid resolution H×W is set to 180×180. Additionally, the default query sizes for the map ($Q_{m}$) and agent queries ($Q_{a}$) are 100×20 and 500, respectively.

During inference at time step *t*, multi-camera images ($I_{t}=\left\{I_{t,1},I_{t,2},\ldots ,I_{t,6}\right\}$, $I_{t,i}\in R^{H\times W\times C}$) are processed through a visual feature extraction network (ResNet-50) to extract features $F_{t}$ corresponding to different camera views:

(4) $$F_{t}=Resnet\left(I_{t}\right)$$

Temporal consistency and scene understanding are enhanced through preservation of preceding BEV features $B_{t-1}$ during inference, maintaining cross-frame feature continuity in the temporal domain. BEV queries $Q_{t}$ are used to decode both the extracted multi-camera features $F_{t}$ and the historical BEV features $B_{t-1}$. This decoding process generates updated BEV queries $B_{t}$ for the current time step, enabling a more comprehensive understanding of the environment. Specifically, the model focuses attention on queries $Q_{sp}$ at different positions in $Q_{t}$ by setting ${\mathrm{Pos}}_{sp}$ through the deformable self-attention mechanism, and then performs attention operations between them and the historical BEV features $B_{t-1}$, allowing different parts of $Q_{t}$ to interact with the historical BEV features to capture the relevant temporal implicit dependencies, generating a time-aware BEV representation $F_{b,temp}$. This mechanism ensures the model effectively integrates historical scene information, improving its perception of the current scene. For instance, objects occluded in the current frame but visible in previous frames can be more accurately detected by leveraging this temporal context:

(5) $$F_{b,temp}=\sum_{Q_{sp}\in Q_{t}}DSA(Q_{sp},{\mathrm{Pos}}_{sp},B_{t-1})$$

where DSA(·) denotes the multi-head deformable self-attention mechanism. Following temporal feature extraction, spatial feature encoding is performed through multi-modal fusion of $Q_{t}$ and multi-camera scene features $F_{t}$, yielding enhanced spatial–temporal representation $F_{b,st}$. This step enhances the model’s spatial understanding of the scene, improving its accuracy in predicting the positions and boundaries of surrounding objects. Notably, by projecting 3D perspectives into a 2D BEV scene through intrinsic and extrinsic camera parameters, we derive the BEV and the mapped positions ${\mathrm{Pos}}_{st}$ for each camera view. These positions ${\mathrm{Pos}}_{st}$, combined with positional encoding from $Q_{t}$, generate corresponding $Q_{st}$ queries. This design ensures that each query $Q_{st}\in Q_{t}$ interacts exclusively with regions of interest in multi-camera views, thereby achieving focused and efficient feature aggregation. This query-based attention mechanism facilitates precise localization and boundary refinement, particularly in complex and cluttered environments:

(6) $$F_{b,st}=\sum_{Q_{st}\in Q_{t}}\sum_{i=1}^{6}CA(Q_{st},{\mathrm{Pos}}_{st},F_{t,i})$$

where CA(·) denotes the multi-head cross-attention mechanism. Subsequent task-specific decoding is performed through dedicated neural decoders, culminating in the derivation of the BEV feature representation $B_{t}$ that encodes the instantaneous environmental topology:

(7) $$B_{t}=CA\left(SA\left(Q_{t}\right),MLP\left(F_{b,st}\right)\right)$$

where SA(·) denotes the multi-head self-attention mechanism. Finally, three lightweight detection heads, map, agent, and traffic, are used to extract high-level semantic information **Info** from $\left(B_{t}\right)$, where each detection head consists of an MLP and has a multi-task output head to extract the corresponding high-level semantic information.

(8) $$\mathrm{Info}=\sum_{c\in \{\mathrm{map},\mathrm{agent},\mathrm{traffic}\}}{\mathrm{MLP}}_{c}\left(\mathrm{CA}\left(\mathrm{SA}\left(B_{t}\right),\left\{F_{b,st},Q_{t}\right\}\right))\left[:,S_{c}\right]\right)$$

where $S_{c}$ represents the allocated feature channel interval. This representation (Info) encapsulates three distinct categories of critical information: (1) Map Information (${\mathrm{Info}}_{m}\in \mathrm{Info}$): This category predicts three types of key map points: road boundaries, lane centers, and lane dividing lines. These predictions collectively form a high-level understanding of the road structure, including lane directions, lane boundary coordinates, and drivable regions. This information is crucial for constructing feasible and safe trajectories. (2) Agent Information (${\mathrm{Info}}_{a}\in \mathrm{Info}$): Agent-related data focus on the motion characteristics of surrounding traffic agents, such as vehicles and pedestrians. This includes their positions, orientations, 3D boundaries, speeds, and future trajectories. By predicting these dynamic attributes, the system enables proactive and adaptive planning in dynamic environments. (3) Traffic Element Information (${\mathrm{Info}}_{e}\in \mathrm{Info}$): Traffic-related elements are integral to safe and efficient driving decisions. This category encompasses signals and signs, such as traffic lights, speed limit signs, and stop signs. Accurate detection and interpretation of these elements guide the vehicle’s compliance with traffic regulations and ensure safe navigation. By combining these three categories of information, Info provides a comprehensive and high-level representation of the driving environment. This serves as the foundation for reliable trajectory planning and robust decision-making in complex and dynamic scenarios.

### 3.4. Bézier Curve-Based Trajectory Planning Module

To overcome the limitations of traditional regression methods for discrete trajectory points, we propose a planning decoder based on Bézier curves. Higher-order Bézier curves inherently ensure continuity in position, velocity, and acceleration. In this work, we adopt cubic Bézier curves, represented by Equation (3), where the control points are generated by our planning head.

In the planning head, a learnable parameter $Q_{ego}$ is initialized to capture scene information essential for trajectory planning. Initially, $Q_{ego}$ interacts with the positions and motion information of other traffic agents through a transformer decoder to extract implicit interaction features:

$$Q_{ego}^{\prime }=Decoder\left(Q_{ego},Q_{a},Q_{a},{\mathrm{Pos}}_{a}\right)$$

here, ${\mathrm{Pos}}_{a}=MLP\left(F_{Infoa}\right)$ provides the positional embedding for traffic agents, where $F_{Infoa}$ represents the decoding feature of ${\mathrm{Info}}_{a}$ and the ego vehicle serves as the BEV coordinate origin and requires no additional position embedding. This interaction enables the model to account for dynamic elements in the driving environment.

Subsequently, the updated $Q_{ego}^{\prime }$ interacts with static traffic elements, such as road infrastructure and traffic signals, to obtain implicit static scene information:

$$Q_{ego}^{\prime \prime }=Decoder\left(Q_{ego}^{\prime },Q_{m},Q_{m},{\mathrm{Pos}}_{m}\right)$$

where ${\mathrm{Pos}}_{m}=MLP\left(F_{Infom},F_{Infoe}\right)$, $F_{Infom}$ and $F_{Infoe}$ represents the decoding feature of ${\mathrm{Info}}_{m}$ and ${\mathrm{Info}}_{e}$. This interaction captures a comprehensive understanding of the static environment, enhancing trajectory planning accuracy in complex driving scenarios.

Finally, the combined features $Q_{ego}^{\prime }$ and $Q_{ego}^{\prime \prime }$, along with discrete navigation information ${\mathrm{Info}}_{n}$ and the ego-vehicle state $s_{ego}$, are input into an MLP-based planning head to compute the control points $P=\{P_{0},P_{1},P_{2},P_{3}\}$ of the Bézier curve:

(9) $$P=PlanHead(Q_{ego}^{\prime },Q_{ego}^{\prime \prime },{\mathrm{Pos}}_{n},s_{ego})$$

where $\left({\mathrm{Pos}}_{n}=MLP\left({\mathrm{Info}}_{n}\right)\right)$. Following the determination of control points P, a continuous Bézier curve is constructed using the Bézier formula, serving as the planned path.

(10) Tbze=∑b=0nnb(1−t)n−btbPb

For practical implementation and control, this continuous curve is then discretized by sampling at uniform time intervals, yielding a sequence of discrete trajectory points $P_{trj}$. These points are utilized by the vehicle’s low-level controller to generate appropriate control commands:

(11) $$T_{bze-d}=\mathrm{Sample}\left(B\right)$$

**Figure 2:**
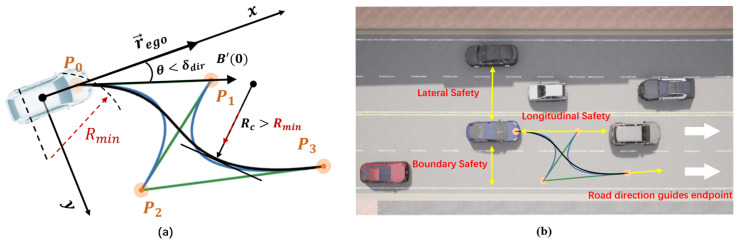
Illustration of planning constraints. (**a**) Visualizes the ego agent’s trajectory planning, constrained by road alignment, driving direction, curvature limits (defined by the minimum turning radius), and trajectory smoothness. (**b**) Depicts the ego agent’s collision avoidance measures, ensuring lateral and longitudinal safety relative to other agents, as well as adherence to road boundary constraints for safe and feasible navigation.

While Bézier curves provide a compact and continuous representation for trajectory planning, challenges remain in ensuring adherence to vehicle kinematics, addressing nonlinear constraints imposed by obstacles, and maintaining effective directional control. To address these issues and ensure that the planned trajectory satisfies kinematic feasibility and driving safety requirements, we introduce a set of constraints, as illustrated in Figure 2. These constraints strategically incorporate the following: (1) Minimum Turning Radius Constraint $C_{k}$, (2) Curvature Continuity Constraint $C_{k_{\max}}$, (3) Collision Avoidance Constraint $C_{\mathrm{col}}$, (4) Road Boundary Adherence Constraint $C_{\mathrm{bod}}$, and (5) Motion Direction Constraint $C_{\mathrm{dir}}$, collectively enforcing the generation of smooth, collision-free, and dynamically feasible trajectories for autonomous navigation systems.

**Minimum Turning Radius Constraint:** The curvature of the trajectory must not exceed a threshold determined by the vehicle’s minimum turning radius ($R_{\min}$) as vehicles have limited steering capabilities. The curvature constraint is defined as

(12) $$|k|>\frac{1}{R_{\min}}\;$$

where $k=\frac{x^{\prime }y^{\prime \prime }-y^{\prime }x^{\prime \prime }}{{\left(x^{\prime 2}+y^{\prime 2}\right)}^{\frac{3}{2}}}$ represents the curvature at each sampling point $T_{bze-d}$ on the Bézier curve trajectory $T_{\mathrm{bze}}$, where (x,y), respectively, represent the coordinate positions of the sampling point in the vehicle’s own coordinate system.

**Curvature Constraint:** The shape of a Bézier curve is dictated by the distribution of its control points. To prevent excessive curvature and ensure smooth trajectories, endpoint curvature constraints and control point smoothness constraints are used to suppress curve curvature:

(13) $$\begin{array}{cc} \frac{2\left|\left(P_{1}-P_{0}\right)\times \left(P_{0}-2P_{1}+P_{2}\right)\right|}{3{\left|P_{1}-P_{0}\right|}^{3}} & +\frac{2\left|\left(P_{3}-P_{2}\right)\times \left(P_{3}-2P_{2}+P_{1}\right)\right|}{3{\left|P_{3}-P_{2}\right|}^{3}}<k_{ep}\\ ∥P_{3}- & 2P_{2}+P_{1}{∥}_{2}<k_{max} \end{array}$$

where $k_{ep}$ is the predefined maximum endpoint curvature threshold and $k_{max}$ is the predefined maximum control point smoothness threshold. This constraint ensures that the control points are appropriately distributed to avoid sharp turns.

**Collision Constraint:** To ensure safety and avoid dangerous interactions with other traffic agents, the trajectory B(t) must maintain a safe distance from all other agents at any future time step t+i, where i≤n. The safety constraint is defined as

$$d_{\mathrm{col}}^{i}>\delta_{\mathrm{col}}$$

Here, $d_{\mathrm{col}}^{i}$ represents the distance between the ego vehicle’s predicted position b(t+i) and the closest traffic participant at time t+i, and $\delta_{\mathrm{col}}$ is a specified safety threshold.

**Road Boundary Constraint:** To prevent collisions with roadside objects and ensure that the trajectory remains within drivable regions, the distance between the planned trajectory points and road boundaries must not exceed a specified threshold:

$$d_{\mathrm{bod}}^{i}<\delta_{\mathrm{bod}},\;\forall i\leq n$$

This constraint ensures the trajectory remains within feasible and safe driving areas.

**Direction Constraint:** The trajectory direction must align with the ego vehicle’s motion direction and comply with road traffic rules. We impose two directional constraints:

(1) At the starting point, the trajectory’s initial direction $B^{\prime }\left(0\right)=3\left(P_{1}-P_{0}\right)$ must align with the ego vehicle’s current direction ${\vec{r}}_{\mathrm{ego}}$:

$$|B^{\prime }\left(0\right)-{\vec{r}}_{\mathrm{ego}}|<\delta_{\mathrm{dir}}$$

(2) At the endpoint, the trajectory’s final direction $B^{\prime }\left(1\right)=3\left(P_{3}-P_{2}\right)$ must align with the road’s driving direction ${\vec{r}}_{\mathrm{road}}$:

$$|B^{\prime }\left(1\right)-{\vec{r}}_{\mathrm{road}}|<\delta_{\mathrm{dir}}$$

## 4. End-to-End Learning

The end-to-end framework is trained using supervised learning. The training process involves separate loss functions for the **scene perception ($L_{\mathrm{SP}}$)** module and the **planning decoder ($L_{\mathrm{PD}}$)** module.

### 4.1. Scene Perception Loss

The scene perception loss focuses on the accuracy of environment perception to ensure the precision of the high-level BEV information Info derived from sensor data. It comprises losses for three categories of predictions: map $L_{\mathrm{map}}$ and agents $L_{\mathrm{agent}}$.

**Map Loss:** The map loss comprises two components: a regression loss and a classification loss. The regression loss evaluates the discrepancy between predicted map points $w_{t}$ and ground truth map points $w_{t}^{gt}$ using an L1 loss. The classification loss assesses the accuracy of map point classifications, where the map categories include ‘Broken’, ‘Solid’, ‘Solid Solid’, and ‘Center’. Traffic elements associated with the map are strictly classified into four categories: ‘traffic sign’, ‘traffic cone’, ‘traffic light’, and ‘others’ using the same loss function. These losses are combined to form the total map loss.

**Agent Loss:** The agent loss supervises the prediction of traffic participant attributes and trajectories using a combination of regression and classification losses. The regression loss ensures the accuracy of predicted location and boundary coordinates of agents. Additionally, the regression loss for trajectory prediction ensures the consistency between predicted trajectory points and actual motion trajectory points. The classification loss supervises the accuracy of agent category predictions, with agents categorized as ’bicycle’, ’car’, ’van’, ’truck’, and ’pedestrian’. These losses are combined into the total agent loss.

The overall scene perception loss combines these components to ensure a comprehensive understanding of the driving environment:

(14) $$L_{\mathrm{SP}}=L_{\mathrm{map}}+L_{\mathrm{agent}}$$

### 4.2. Planning Decoder Loss

The planning decoder loss ensures the accuracy and safety of ego vehicle trajectory predictions. In our approach, the predictions are based on a relative coordinate system centered on the ego vehicle rather than an absolute map-based coordinate system. The onboard sensors provide feedback on the vehicle’s velocity, acceleration, and GPS position.

The planning decoder loss consists of two parts: (1). Bézier curve constraints $L_{bez}$: supervises adherence to trajectory constraints. (2). Trajectory prediction loss ($L_{\mathrm{traj}}$): L1 loss between the predicted and ground truth trajectories.

**Bézier Curve Constraints:** The Bézier curve constraint architecture, analogous to those in Section 3.4, is systematically structured into five components to ensure physical feasibility and safety compliance. The Minimum Turning Radius Constraint ($C_{Rmin}$) ensures the curvature of the trajectory does not exceed the physical minimum turning radius. The curvature constraint ($C_{cc}$) penalizes trajectories with curvatures and smoothness exceeding the predefined maximum allowable threshold. Road boundary adherence is enforced through the boundary constraint ($C_{\mathrm{bod}}$). Collision avoidance is guaranteed by the collision constraint ($C_{\mathrm{col}}$). Directional consistency is achieved via the composite constraint $C_{\mathrm{dir}}$, which quantifies alignment between the initial trajectory derivative and the ego vehicle’s heading and evaluates terminal directional deviation from the road’s orientation. These constraints all set penalty losses that increase as thresholds are exceeded, and they are jointly optimized to generate kinematically acceptable trajectories.

**Trajectory Prediction Loss:** The L1 loss function quantifies the deviations between predicted Bézier trajectories and their ground-truth counterparts by computing the L1-norm distance across trajectory coordinates.

The overall planning decoder loss is formulated as

(15) $$L_{\mathrm{PD}}=L_{bez}+L_{\mathrm{traj}}$$

The proposed loss functions ensure precise perception and safe trajectory planning. $L_{\mathrm{SP}}$ guarantees high-level environmental understanding by supervising map, agent, and traffic element predictions. $L_{\mathrm{PD}}$ enforces kinematic feasibility and compliance with safety constraints, achieving smooth, reliable, and accurate trajectory generation. This robust loss design underpins the effectiveness of our end-to-end autonomous driving framework.

## 5. Experiments

This section methodologically presents our experimental framework, visualizes key findings, and provides systematic analysis through three structured components: **Implementation Details**, **Results**, and **Ablation Study**.

### 5.1. Implementation Details

Comprehensive evaluations were conducted on the nuScenes [50] benchmark dataset, which contains 1000 unique driving scenarios, with each sequence spanning approximately 20 s. The dataset provides 1.4 million meticulously annotated 3D bounding boxes across 23 distinct object categories. The visual data were captured through a synchronized six-camera array (front, front-left, front-right, back, back-left, and back-right), achieving full 360° environmental coverage, with critical frames annotated at 2 Hz temporal resolution. Following established evaluation protocols in prior research, we employed two principal metrics for planning performance assessment: (1) displacement error, measuring the L2 distance between predicted and ground-truth trajectories at specified timestamps; and (2) collision rate, quantifying the percentage of trajectory predictions involving potential vehicle collisions. In addition, to validate the generalization capability of the proposed method, we conducted a set of simplified generalization tests using the curated scenario library from the Bench2Drive [51] benchmark.

The proposed framework processes a 2-s historical observation window to generate 3-second predictive trajectories. The architecture employs a pre-trained ResNet50 backbone for multi-scale feature extraction from six camera inputs. The perception system operates within a 60 m (longitudinal) × 30 m (lateral) operational domain, implementing simultaneous vector mapping and motion prediction through our novel multi-task learning framework. Input images are standardized to 640 × 360 resolution with photometric normalization applied during preprocessing. The complete system required 80 training epochs (168 GPU-hours) on an 8× NVIDIA RTX 3090 (24 GB VRAM per GPU) using distributed data-parallel strategy with synchronized batch normalization.

### 5.2. Results

**Figure 3:**
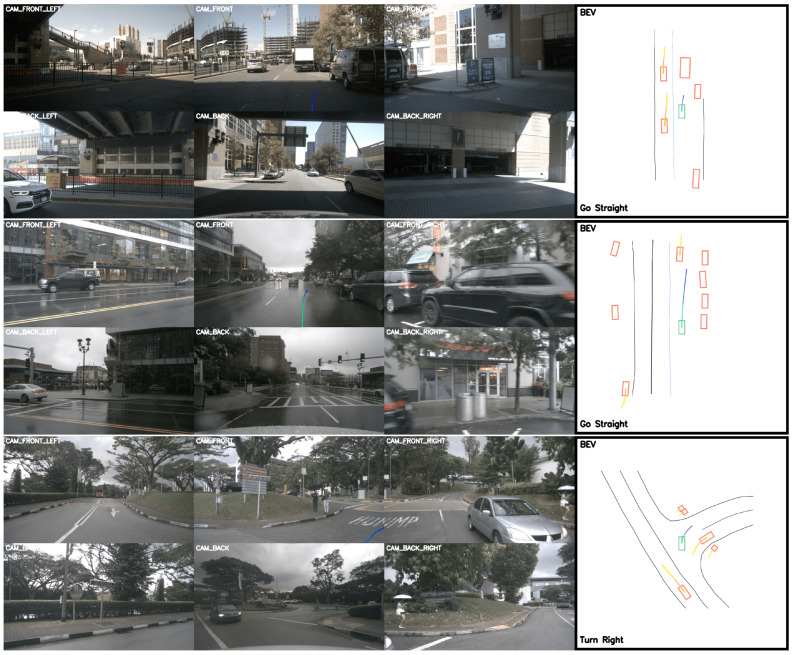
Visualization tested on the nuScenes benchmark.

Comprehensive benchmarking against outstanding baselines was conducted on the nuScenes dataset, with quantitative comparisons systematically documented in the results in Table 1, and the visualization images are displayed in Figure 3. The compared methods are as follows:

1. NMP [52]: a holistic neural motion planning framework processes raw LiDAR data and HD maps to generate interpretable 3D detections, trajectory predictions, and a multi-modal cost volume encoding navigational preferences. It selects optimal trajectories by sampling physically feasible candidates and minimizing the learned cost function that inherently captures diverse driving scenario dynamics.
2. FF [53]: a self-supervised learning framework for motion planning, bypassing object-centric scene representations by directly modeling occupancy dynamics through automatically generated freespace boundaries. The approach integrates two novel components: (1) collision-prone trajectory identification via freespace violation analysis in planning horizons, and (2) annotation-free supervision through differentiable freespace constraints in neural planner training pipelines.
3. EO [54]: a motion-invariant scene representation for self-supervised trajectory forecasting using differentiable raycasting to project predicted 3D occupancy into future LiDAR sweep predictions for training. By rendering unobserved regions (occlusions) through neural volumetric reasoning, occupancy inherently disentangles ego motion from dynamic environment changes, enabling direct integration with planners to avoid non-drivable regions.
4. ST-P3 [10]: an interpretable vision-based end-to-end framework (ST-P3) that jointly learns spatial–temporal features for perception, prediction, and planning through three core components: egocentric-aligned 3D geometry preservation before perception, dual-pathway motion history modeling for trajectory forecasting, and temporal refinement to enhance vision-centric planning cues. It unifies scene understanding, dynamics inference, and control reasoning within a single coherent pipeline by explicitly addressing geometry distortion, motion context aggregation, and visual feature adaptation across tasks.
5. UniAD [13]: a planning-oriented unified framework that integrates full-stack driving tasks (perception, prediction, and interaction modeling) into a single network, prioritizing feature abstraction and inter-task synergy through shared queries and complementary module designs. It establishes task communication via unified interfaces to jointly optimize scene understanding, behavior reasoning, and trajectory generation in a globally coordinated manner for end-to-end planning.
6. VAD [46]: an end-to-end vectorized autonomous driving framework that models driving scenes with fully vectorized representations, achieving improved safety through instance-level planning constraints and significantly faster computation compared to raster-based methods while also attaining strong baseline planning performance on the nuScenes dataset.

The experimental results demonstrate significant improvements in both trajectory accuracy and safety metrics. Our method achieves superior performance across all time horizons, with an average L2 displacement error of 0.49 m (38.3% lower than VAD-Base’s 0.72 m) and an average collision rate of 0.16% (27.3% reduction from VAD-Base’s 0.22%). Notably, the Bézier-parameterized trajectory framework shows remarkable consistency in long-term predictions, maintaining a minimal error growth from 0.45 m at 1 s to 0.54 m at 3 s, significantly outperforming conventional methods that exhibit error accumulation over time (e.g., FF increases from 0.55 m to 2.54 m). The collision rate progression (0.04% → 0.11% → 0.33%) reveals enhanced safety characteristics compared to the baseline approaches, validating our query-based interaction mechanism’s effectiveness in capturing critical environmental constraints. Particularly noteworthy is the method’s performance in 3 s predictions—the most challenging horizon—where we achieve 51.4% lower L2 error and 19.5% lower collision rate than the prior state-of-the-art approach (VAD-Base). These improvements confirm our framework’s ability to maintain kinematic feasibility through Bézier curve parameterization while ensuring robust perception through explicit feature fusion. The results validate our design objectives of achieving smooth trajectory transitions and improved interpretability in complex driving scenarios.

**Figure 4:**
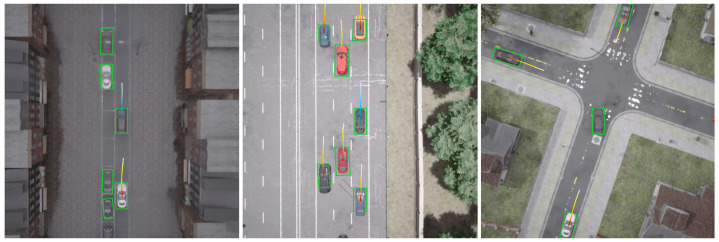
Visualization images of generalization testing.

The generalization capability was evaluated through three composite scenarios constructed from the Bench2Drive benchmark: **(1) static hazard scenarios:** designed to assess vehicle performance when encountering stationary obstacles or immobile hazards, comprising ParkedObstacle, ConstructionObstacle, ConstructionObstacleTwoWays, Accident, AccidentTwoWays, and HazardAtSideLane; **(2) dynamic interaction scenarios:** testing trajectory conflict resolution with moving agents, including ParkingCutIn, HighwayCutIn, MergerIntoSlowTraffic, EnterActorFlow, ParkingExit, and VehicleTurningRoutePedestrian; and **(3) regulated junction scenarios:** evaluating intersection operations under regulatory constraints, with sub-scenarios such as SignalizedJunctionRightTurn, VehicleTurningRoute, VanillaSignalizedTurnEncounterRedLight, NonSignalizedJunctionLeftTurn, NonSignalizedJunctionRightTurn, and VanillaNonSignalizedTurn. The driving performance was evaluated using three key metrics: (1) driving score: calculated as the aggregated mean score across all the routes within each scenario, reflecting comprehensive driving quality; (2) route completion: measured by the averaged percentage of successfully navigated route segments per scenario; and (3) infraction score: initialized at 1.0 and subject to proportional discounting upon traffic rule violations, with the final values representing the normalized compliance levels across all the scenario routes. The experimental results are presented in Table 2, and the visualization is shown in Figure 4.

The generalization tests demonstrate our framework’s scenario-adaptive capabilities. The superior performance in dynamic interaction scenarios (95.46% route completion) validates the effectiveness of Bézier-parameterized trajectories in handling agent motion uncertainties, while the consistent infraction scores (average 0.64) across all the scenarios prove the safety benefits from the query-based multi-modal fusion. Notably, the 99% completion rate in regulated junctions confirms the BEV-Transformer’s interpretable spatial reasoning ability for structured environments. However, the relatively low driving score in static hazards (37.00) suggests that pure vision-based perception still faces challenges in severe occlusion scenarios, which could be mitigated by integrating complementary sensors in future work. These results collectively substantiate our core claim of achieving enhanced perception-planning synergy through unified BEV encoding and continuous trajectory optimization.

### 5.3. Ablation Study

Ablation studies were conducted to validate the individual contributions of methodological components by sequentially deactivating the following modules: map query, agent query (including its ego-specific submodule), BEV query, and Bézier curve constraints.

Ablation studies conducted on key components of our framework reveal critical insights into their individual contributions to system performance. As shown in Table 3, removing map queries increases the average L2 error by 10.2% (0.54 vs. 0.49) and collision rate by 43.8% (0.23 vs. 0.16), demonstrating the essential role of explicit environmental encoding in perception accuracy. The absence of agent queries degrades performance most severely, with L2 error doubling (1.04 vs. 0.49) and collision rate increasing by 62.5%, confirming the necessity of modeling traffic participant interactions. Notably, eliminating ego-vehicle queries causes disproportionate collision rate degradation (0.32 vs. 0.16), suggesting this component’s critical role in self-awareness for collision avoidance. The BEV query removal experiment shows comparable L2 degradation to map query removal (0.56 vs. 0.54) but yields better collision metrics, implying that BEV features primarily enhance spatial localization rather than interactive safety. The Bézier parameterization ablation increases L2 error by 63.3% (0.80 vs. 0.49) while maintaining collision performance comparable to map query removal, validating its effectiveness in trajectory smoothing and kinematic feasibility. The cumulative degradation when removing all the query mechanisms (L2: 0.99 and collision: 0.32) highlights the synergistic effects of our multi-modal interaction design. Our complete framework achieves optimal balance between trajectory accuracy (0.49 L2) and safety (0.16 collisions), proving the necessity of integrated perception-planning components for robust autonomous navigation.

## 6. Conclusions

End-to-end autonomous driving demands precise perception, robust motion planning, and efficient trajectory generation to navigate complex and dynamic environments. This paper proposes BETAV, a novel framework that addresses the persistent challenges of low 3D perception accuracy and suboptimal trajectory smoothness in autonomous driving systems. The proposed framework, integrating Vision-Transformer-based BEV perception and Bézier curve-based trajectory planning, demonstrates significant advancements in autonomous driving systems by achieving state-of-the-art trajectory accuracy and collision avoidance performance. The query-based interaction mechanism and compact Bézier representation effectively address the challenges of multi-modal fusion and motion smoothness, while explicit kinematic constraints ensure safety across diverse scenarios. However, the current method exhibits limitations in computational efficiency due to the reliance on transformer architectures for real-time BEV encoding, which may hinder deployment on resource-constrained platforms. Additionally, Bézier parameterization, while simplifying trajectory optimization, could face challenges in highly dynamic environments with abrupt behavioral changes as its continuity assumptions may limit adaptability to extreme maneuvers.

Future work will focus on optimizing the framework’s computational footprint through lightweight transformer variants or hybrid architectures combining CNNs and attention mechanisms. Enhancing the Bézier decoder with adaptive curvature constraints and integrating uncertainty-aware planning modules could further improve robustness in unpredictable scenarios. Extending the framework to incorporate real-world traffic rule reasoning and multi-agent interaction modeling will also be critical for achieving human-like decision-making. Finally, validation in open-world environments with long-tail edge cases and diverse sensor configurations remains essential to bridge the gap between simulation-based performance and practical deployment. Addressing these challenges will advance the development of safer, more generalizable autonomous driving systems.

## Figures and Tables

**Table 1:** Performance comparison on motion planning metrics.

Method	L2 (m)				Collision (%)			
	**1 s**	**2 s**	**3 s**	**Avg.**	**1 s**	**2 s**	**3 s**	**Avg.**
NMP [52]	–	–	2.31	–	–	–	1.92	–
SA-NMP [52]	–	–	2.05	–	–	–	1.59	–
FF [53]	0.55	1.20	2.54	1.43	0.06	0.17	1.07	0.43
EO [54]	0.67	1.36	2.78	1.60	0.04	0.09	0.88	0.33
ST-P3 [10]	1.33	2.11	2.90	2.11	0.23	0.62	1.27	0.71
UniAD [13]	0.48	0.96	1.65	1.03	0.05	0.17	0.71	0.31
VAD-Tiny [46]	0.46	0.76	1.12	0.78	0.21	0.35	0.58	0.38
VAD-Base [46]	0.41	0.70	1.05	0.72	0.07	0.17	0.41	0.22
Ours	0.45	0.48	0.54	0.49	0.04	0.11	0.33	0.16

**Table 2:** Generalization testing across multiple scenarios.

Scenarios	Driving Score	Route Completion	Infraction Score
Static Hazard	37.00	52.63	0.67
Dynamic Interaction	69.16	95.46	0.72
Regulated Junction	53.28	99.00	0.53
Average	51.47	78.99	0.64

**Table 3:** Ablation study of different components.

w/o $Q_{m}$	w/o $Q_{a}$	w/o $Q_{\mathrm{ego}}$	w/o $Q_{t}$	w/o $C_{b}$	L2 (Avg.)	Collision (Avg.)
✓	-	-	-	-	0.54	0.23
-	✓	-	-	-	1.04	0.26
-	-	✓	-	-	0.98	0.32
-	-	-	✓	-	0.56	0.20
-	-	-	-	✓	0.80	0.26
✓	✓	✓	✓	-	0.99	0.32
-	-	-	-	-	0.49	0.16

## Data Availability

The data presented in this study are available upon request from the corresponding author.

## References

[1] Parekh D., Poddar N., Rajpurkar A., Chahal M., Kumar N., Joshi G.P., Cho W. (2022). A review on autonomous vehicles: Progress, methods and challenges. Electronics.

[2] Garikapati D., Shetiya S.S. (2024). Autonomous vehicles: Evolution of artificial intelligence and the current industry landscape. Big Data Cogn. Comput..

[3] Padmaja B., Moorthy C.V., Venkateswarulu N., Bala M.M. (2023). Exploration of issues, challenges and latest developments in autonomous cars. J. Big Data.

[4] Chen L., Platinsky L., Speichert S., Osiński B., Scheel O., Ye Y., Grimmett H., Del Pero L., Ondruska P. (2021). What data do we need for training an av motion planner?. Proceedings of the 2021 IEEE International Conference on Robotics and Automation (ICRA).

[5] González D., Pérez J., Milanés V., Nashashibi F. (2015). A review of motion planning techniques for automated vehicles. IEEE Trans. Intell. Transp. Syst..

[6] Kendall A., Hawke J., Janz D., Mazur P., Reda D., Allen J.M., Lam V.D., Bewley A., Shah A. (2019). Learning to drive in a day. Proceedings of the 2019 International Conference on Robotics and Automation (ICRA).

[7] Xu W., Wang Q., Dolan J.M. (2021). Autonomous vehicle motion planning via recurrent spline optimization. Proceedings of the 2021 IEEE International Conference on Robotics and Automation (ICRA).

[8] Casas S., Sadat A., Urtasun R. Mp3: A unified model to map, perceive, predict and plan. Proceedings of the IEEE/CVF Conference on Computer Vision and Pattern Recognition.

[9] Cui A., Casas S., Sadat A., Liao R., Urtasun R. Lookout: Diverse multi-future prediction and planning for self-driving. Proceedings of the IEEE/CVF International Conference on Computer Vision.

[10] Hu S., Chen L., Wu P., Li H., Yan J., Tao D. (2022). St-p3: End-to-end vision-based autonomous driving via spatial-temporal feature learning. Proceedings of the European Conference on Computer Vision.

[11] Chib P.S., Singh P. (2023). Recent advancements in end-to-end autonomous driving using deep learning: A survey. IEEE Trans. Intell. Veh..

[12] Chen L., Wu P., Chitta K., Jaeger B., Geiger A., Li H. (2024). End-to-end autonomous driving: Challenges and frontiers. IEEE Trans. Pattern Anal. Mach. Intell..

[13] Hu Y., Yang J., Chen L., Li K., Sima C., Zhu X., Chai S., Du S., Lin T., Wang W. Planning-oriented autonomous driving. Proceedings of the IEEE/CVF Conference on Computer Vision and Pattern Recognition.

[14] Raghu M., Unterthiner T., Kornblith S., Zhang C., Dosovitskiy A. (2021). Do vision transformers see like convolutional neural networks?. Adv. Neural Inf. Process. Syst..

[15] Guo J., Han K., Wu H., Tang Y., Chen X., Wang Y., Xu C. Cmt: Convolutional neural networks meet vision transformers. Proceedings of the IEEE/CVF Conference on Computer Vision and Pattern Recognition.

[16] Yang K., Zhong M., Fan K., Tan J., Xiao Z., Deng Z. Multi-Task Traffic Scene Perception Algorithm Based on Multi-Scale Prompter. https://papers.ssrn.com/sol3/papers.cfm?abstract_id=4965636.

[17] Lei Y., Wang Z., Chen F., Wang G., Wang P., Yang Y. (2023). Recent advances in multi-modal 3d scene understanding: A comprehensive survey and evaluation. arXiv.

[18] Khanam R., Hussain M. (2024). Yolov11: An overview of the key architectural enhancements. arXiv.

[19] Duan K., Bai S., Xie L., Qi H., Huang Q., Tian Q. Centernet: Keypoint triplets for object detection. Proceedings of the IEEE/CVF International Conference on Computer Vision.

[20] Backhaus D., Engbert R., Rothkegel L.O., Trukenbrod H.A. (2020). Task-dependence in scene perception: Head unrestrained viewing using mobile eye-tracking. J. Vis..

[21] Liu W., Hua M., Deng Z., Meng Z., Huang Y., Hu C., Song S., Gao L., Liu C., Shuai B. (2023). A systematic survey of control techniques and applications in connected and automated vehicles. IEEE Internet Things J..

[22] Liang J., Yang K., Tan C., Wang J., Yin G. (2025). Enhancing High-Speed Cruising Performance of Autonomous Vehicles Through Integrated Deep Reinforcement Learning Framework. IEEE Trans. Intell. Transp. Syst..

[23] Liang J., Tian Q., Feng J., Pi D., Yin G. (2024). A Polytopic Model-Based Robust Predictive Control Scheme for Path Tracking of Autonomous Vehicles. IEEE Trans. Intell. Veh..

[24] Zhang H., Zhao Z., Wei Y., Liu Y., Wu W. (2025). A self-tuning variable universe fuzzy PID control framework with hybrid BAS-PSO-SA optimization for unmanned surface vehicles. J. Mar. Sci. Eng..

[25] Van N.D., Sualeh M., Kim D., Kim G.W. (2020). A hierarchical control system for autonomous driving towards urban challenges. Appl. Sci..

[26] Lan G., Hao Q. (2023). End-to-end planning of autonomous driving in industry and academia: 2022-2023. arXiv.

[27] Ji H., Liang P., Cheng E. Enhancing 3D object detection with 2D detection-guided query anchors. Proceedings of the IEEE/CVF Conference on Computer Vision and Pattern Recognition.

[28] Wang Y., Guizilini V.C., Zhang T., Wang Y., Zhao H., Solomon J. Detr3d: 3d object detection from multi-view images via 3D-to-2D queries. Proceedings of the Conference on Robot Learning.

[29] Liu Y., Wang T., Zhang X., Sun J. (2022). Petr: Position embedding transformation for multi-view 3d object detection. Proceedings of the European Conference on Computer Vision.

[30] Vaswani A., Shazeer N., Parmar N., Uszkoreit J., Jones L., Gomez A.N., Kaiser L., Polosukhin I. Attention is all you need. Proceedings of the Advances in Neural Information Processing Systems 30: Annual Conference on Neural Information Processing Systems 2017.

[31] Chen S., Cheng T., Wang X., Meng W., Zhang Q., Liu W. (2022). Efficient and robust 2d-to-bev representation learning via geometry-guided kernel transformer. arXiv.

[32] Hu A., Murez Z., Mohan N., Dudas S., Hawke J., Badrinarayanan V., Cipolla R., Kendall A. Fiery: Future instance prediction in bird’s-eye view from surround monocular cameras. Proceedings of the IEEE/CVF International Conference on Computer Vision.

[33] Li Z., Wang W., Li H., Xie E., Sima C., Lu T., Yu Q., Dai J. (2022). BEVFormer: Learning Bird’s-Eye-View Representation from Multi-Camera Images via Spatiotemporal Transformers. arXiv.

[34] Liao B., Chen S., Jiang B., Cheng T., Zhang Q., Liu W., Huang C., Wang X. (2024). Lane graph as path: Continuity-preserving path-wise modeling for online lane graph construction. Proceedings of the European Conference on Computer Vision.

[35] Liao B., Chen S., Wang X., Cheng T., Zhang Q., Liu W., Huang C. (2022). Maptr: Structured modeling and learning for online vectorized hd map construction. arXiv.

[36] Liu Z., Chen S., Guo X., Wang X., Cheng T., Zhu H., Zhang Q., Liu W., Zhang Y. Vision-based uneven bev representation learning with polar rasterization and surface estimation. Proceedings of the Conference on Robot Learning, PMLR.

[37] Zhang Y., Zhu Z., Zheng W., Huang J., Huang G., Zhou J., Lu J. (2022). Beverse: Unified perception and prediction in birds-eye-view for vision-centric autonomous driving. arXiv.

[38] Philion J., Fidler S. (2020). Lift, splat, shoot: Encoding images from arbitrary camera rigs by implicitly unprojecting to 3d. Proceedings of the Computer Vision—ECCV 2020: 16th European Conference.

[39] Roddick T., Kendall A., Cipolla R. (2018). Orthographic feature transform for monocular 3d object detection. arXiv.

[40] Rukhovich D., Vorontsova A., Konushin A. Imvoxelnet: Image to voxels projection for monocular and multi-view general-purpose 3d object detection. Proceedings of the IEEE/CVF Winter Conference on Applications of Computer Vision.

[41] Xie E., Yu Z., Zhou D., Philion J., Anandkumar A., Fidler S., Luo P., Alvarez J.M. (2022). M^2^ BEV: Multi-camera joint 3D detection and segmentation with unified birds-eye view representation. arXiv.

[42] Codevilla F., Santana E., López A.M., Gaidon A. Exploring the limitations of behavior cloning for autonomous driving. Proceedings of the IEEE/CVF International Conference on Computer Vision.

[43] Prakash A., Chitta K., Geiger A. Multi-modal fusion transformer for end-to-end autonomous driving. Proceedings of the IEEE/CVF Conference on Computer Vision and Pattern Recognition.

[44] Sadat A., Casas S., Ren M., Wu X., Dhawan P., Urtasun R. (2020). Perceive, predict, and plan: Safe motion planning through interpretable semantic representations. Proceedings of the Computer Vision—ECCV 2020: 16th European Conference.

[45] Renz K., Chitta K., Mercea O.B., Koepke A., Akata Z., Geiger A. (2022). Plant: Explainable planning transformers via object-level representations. arXiv.

[46] Jiang B., Chen S., Xu Q., Liao B., Chen J., Zhou H., Zhang Q., Liu W., Huang C., Wang X. Vad: Vectorized scene representation for efficient autonomous driving. Proceedings of the IEEE/CVF International Conference on Computer Vision.

[47] Han Z., Wu Y., Li T., Zhang L., Pei L., Xu L., Li C., Ma C., Xu C., Shen S. (2023). An efficient spatial-temporal trajectory planner for autonomous vehicles in unstructured environments. IEEE Trans. Intell. Transp. Syst..

[48] Chen W., Chen Y., Wang S., Kong F., Zhang X., Sun H. (2023). Motion planning using feasible and smooth tree for autonomous driving. IEEE Trans. Veh. Technol..

[49] Chen S., Jiang B., Gao H., Liao B., Xu Q., Zhang Q., Huang C., Liu W., Wang X. (2024). Vadv2: End-to-end vectorized autonomous driving via probabilistic planning. arXiv.

[50] Caesar H., Bankiti V., Lang A.H., Vora S., Liong V.E., Xu Q., Krishnan A., Pan Y., Baldan G., Beijbom O. nuScenes: A multimodal dataset for autonomous driving. Proceedings of the IEEE/CVF Conference on Computer Vision and Pattern Recognition.

[51] Jia X., Yang Z., Li Q., Zhang Z., Yan J. (2024). Bench2drive: Towards multi-ability benchmarking of closed-loop end-to-end autonomous driving. arXiv.

[52] Zeng W., Luo W., Suo S., Sadat A., Yang B., Casas S., Urtasun R. End-to-end interpretable neural motion planner. Proceedings of the IEEE/CVF Conference on Computer Vision and Pattern Recognition.

[53] Hu P., Huang A., Dolan J., Held D., Ramanan D. Safe local motion planning with self-supervised freespace forecasting. Proceedings of the IEEE/CVF Conference on Computer Vision and Pattern Recognition.

[54] Khurana T., Hu P., Dave A., Ziglar J., Held D., Ramanan D. (2022). Differentiable raycasting for self-supervised occupancy forecasting. Proceedings of the European Conference on Computer Vision.

